# A Cost‐Effective and Scalable Machine Learning Approach for Quality Assessment of Fresh Maize Kernel Using NIR Spectroscopy

**DOI:** 10.1002/advs.202512750

**Published:** 2025-09-29

**Authors:** Jiang Shi, Erkui Yue, Xuejin Zhu, Lin Zhao, Weifeng Chen, Xiangqun Yu, Hongliang Huang, Ying Qian, Zhengfang Zhang, Jianguo Wu

**Affiliations:** ^1^ Institute of Crop and Ecology Hangzhou Academy of Agricultural Sciences Hangzhou 310024 P. R. China; ^2^ College of Science Hangzhou Dianzi University Hangzhou 310018 P. R. China; ^3^ School of Information Zhejiang University of Finance and Economics Hangzhou 310018 P. R. China; ^4^ Shaoxing Keqiao District Agricultural Technology Training School Shaoxing 312031 P. R. China; ^5^ College of Horticulture Science Zhejiang A&F University Hangzhou 311300 P. R. China

**Keywords:** fresh maize kernel, near‐infrared spectroscopy, prediction‐correction neural network, small sample set, scalability

## Abstract

In fresh maize breeding, developing robust and accurate near‐infrared (NIR) calibration models traditionally requires significant time, cost, and labor. To address these challenges, a novel machine learning approach is proposed using a Prediction‐Correction Neural Network (PCNN) that enables effective modeling from small sample sets augmented with synthetic data based on NIR spectroscopy. For key quality traits such as amylopectin, protein, crude fiber, and total sugar, the PCNN achieved residual predictive deviation (RPD) values between 2.821 and 4.862, and coefficients of determination ($R_{V}^{2}$) ranging from 0.869 to 0.951, using an average of only 32 calibration samples. For sugars including fructose, glucose, and sucrose, the model yielded RPD >2 and $R_{V}^{2}\geq 0.747$ with just 62 samples. The PCNN method has also been successfully applied to NIR model development for small sample sets in intact kernel of fresh maize and other crops, including forage maize, rice, wheat, and barley. Compared to Partial Least Squares (PLS) and traditional Artificial Neural Networks (ANN), PCNN delivered RPD improvements of 38.99%−63.20% over PLS and 7.07%−25.82% over ANN. These results highlight the PCNN's high efficiency and accuracy, offering a scalable and cost‐effective solution for rapid quality evaluation in fresh maize and other cereals.

## Graphical abstract

A cost‐effective and scalable PCNN approach is developed for NIR model construction that has compatibility with multiple instruments and multiple crops. It is time and cost saving, rapid and nondestructive, small sample set based, various quality properties and extensible for breeding application in fresh maize, forage maize, rice, wheat, and barley.

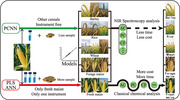

## 1 Introduction

Fresh maize (*Zea mays* L.) is deliberately harvested before full maturity to preserve its distinctive flavor and texture. Compared to traditional maize varieties, fresh maize is characterized by more tender kernels, higher sugar and amylopectin content, lower crude fiber, and enhanced taste and nutritional values, making it particularly popular among consumers.^[^ ^1^, ^2^ ^]^ In China, fresh maize encompasses several types, including sweet maize, waxy maize, and sweet‐waxy maize.^[^ ^3^ ^]^ Key components that influence the sensory qualities of fresh maize – such as texture and flavor – include total sugars (fructose, glucose, sucrose), amylopectin, protein, and crude fiber.^[^ ^1^, ^2^, ^4^ ^]^ However, due to its high moisture content, fresh maize deteriorates quickly after harvest and is unsuitable for long‐term storage.^[^ ^1^ ^]^ Current methods for evaluating kernel quality rely heavily on chemical analyses, which are labor‐intensive, time‐consuming, and often require expensive and destructive reagents.^[^ ^5^, ^6^ ^]^ Consequently, there is an urgent need for a rapid, simple, and environmentally friendly method to assess the key quality components of fresh maize, supporting applications in high‐quality breeding, commercial quality control, and food processing.

NIR spectroscopy is a rapid, nondestructive analytical technique widely used to assess the physicochemical properties of maize. It operates based on the absorption characteristics of materials in the near‐infrared region (800−2500 nm).^[^ ^7^ ^]^ Most existing studies have focused on predicting components in mature maize, such as protein, oil, and starch content in kernels,^[^ ^8^, ^9^ ^]^ as well as oil‐related traits in single kernels.^[^ ^10^, ^11^ ^]^ Similarly, NIR applications in fresh maize have primarily targeted mature seeds – for instance, in distinguishing sweet maize varieties,^[^ ^12^ ^]^ measuring starch and amylose in waxy maize,^[^ ^13^ ^]^ and evaluating seed vigor in sweet maize.^[^ ^14^, ^15^ ^]^ However, few studies have explored NIR applications in filling‐stage kernels of fresh maize. Existing research has investigated components such as soluble sugars,^[^ ^16^, ^17^ ^]^ protein,^[^ ^18^ ^]^ and moisture content,^[^ ^19^, ^20^ ^]^ but these typically rely on large sample sets and require time‐consuming, labor‐intensive wet chemistry for calibration. Additionally, most models are case‐specific and tied to a single NIR instrument. Therefore, there is a critical need to develop a generalized NIR calibration model that is effective with small sample sets and compatible across different spectrometers and cereal types.

Despite its advantages, NIR spectroscopy faces several challenges, including the nonlinear behavior of spectral absorbance,^[^ ^21^ ^]^ wavelength selection,^[^ ^22^ ^]^ the identification of representative samples,^[^ ^23^ ^]^ variability in physicochemical properties,^[^ ^24^ ^]^ and limitations related to the size of the calibration dataset.^[^ ^25^ ^]^ To address these complexities, machine learning—an advanced, data‐driven branch of artificial intelligence—offers powerful tools capable of modeling both linear and nonlinear relationships between high‐dimensional input data and target outputs.^[^ ^26^ ^]^ Among these, ANN is inspired by the structure and function of the human brain and excel at learning complex patterns and predictive relationships.^[^ ^27^, ^28^, ^29^ ^]^ When integrated with NIR spectroscopy, ANNs can effectively model and predict quality traits in maize kernels, including sweetness, waxiness, and nutritional content. While most studies have relied on large datasets to train such models, there is growing interest in developing robust and accurate models using small sample sets with reference data – a more efficient and practical approach for many real‐world applications.^[^ ^19^, ^20^ ^]^

To address the challenges outlined above, the PCNN method was developed. This approach leverages a small number of calibration samples while retaining the powerful nonlinear modeling capabilities of ANN. The PCNN framework consists of two independent neural networks: a Prediction Neural Network (PNN) and a Correction Neural Network (CNN). The PNN is trained using synthetic sample data, enabling it to predict component value for both calibration and validation sets. These synthetic samples are generated by assigning standard pure components to mimic real maize kernel compositions. The CNN is then trained to correct the discrepancies between the PNN's predictions and the actual reference values in the calibration set. Final component estimations are obtained by combining the outputs from both the prediction and correction networks, resulting in more accurate and reliable predictions.

**Figure 1:**
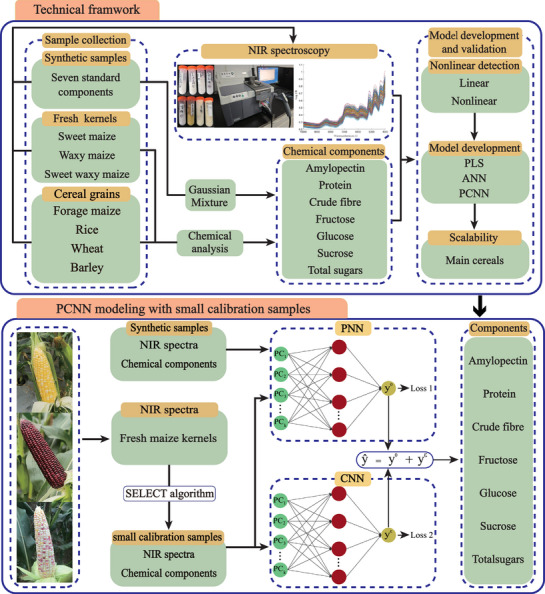
Technical framework of cost‐competitive quality evaluation of fresh maize kernels. The workflow includes sample collection, chemical reference analysis, NIR spectra acquisition, model development, and validation. A novel PCNN method is introduced to predict seven key components of fresh maize kernels using a small calibration dataset.

Using the PCNN method, seven key components were predicted: amylopectin, protein, crude fiber, fructose, glucose, sucrose, and total sugar. The process consisted of several key steps. First, NIR spectral data were collected using a spectrophotometer from various grain samples, including sweet maize, waxy maize, sweet–waxy maize, forage maize, as well as rice, wheat, barley, and synthetic samples of the seven standard components (**Figure** **1**). Second, the nonlinear relationships between NIR spectra and component concentrations in fresh maize were assessed using the Durbin–Watson (D‐W) statistic and runs tests. Third, for datasets with abundant samples, component predictions were performed using both PLS regression and a fully connected feed‐forward ANN with a single hidden layer, allowing for a comparison of predictive performance between the ANN and the traditional PLS approach. Fourth, for small sample sets, the PCNN method – enhanced with synthetic sample integration – was applied. Model parameters for the prediction and correction networks were optimized using two distinct loss functions (*Loss*1 and *Loss*2) with the Adam optimizer. Two sampling strategies were evaluated: the SELECT method (based on spectral characteristics) and random selection. Optimal sample size was determined using the neighborhood (NH) Mahalanobis distance metric. Finally, the PCNN method was extended to construct protein prediction models for forage maize, rice, wheat, and barley. Comparative analysis with PLS and ANN models demonstrated the PCNN's superior performance and its potential for broader application in cereal quality assessment.

## 2 Data Acquisition and Preprocessing

### 2.1 Sample Collection and Preparation

**Figure 2:**
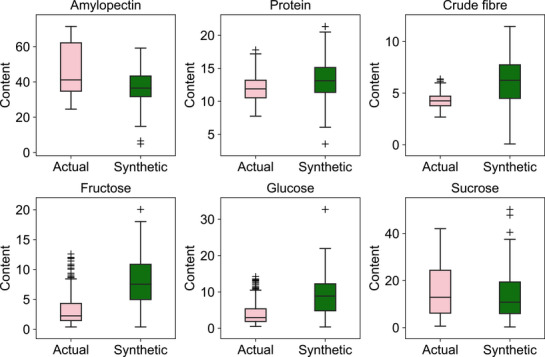
Box plots of component distributions in fresh maize kernels and synthetic standard samples. Pink box plots represent the seven components measured from 246 fresh maize kernel samples, while green box plots represent those of 99 synthetic standard samples.

To develop a robust and reliable NIR calibration model for fresh maize kernels, a total of 246 maize varieties were collected in July 2022 from the experimental base of the Hangzhou Academy of Agricultural Sciences, Zhejiang Province. These included 140 sweet maize, 72 waxy maize, and 34 sweet‐waxy maize varieties. At the kernel filling stage (21−23 days after pollination), 4−5 ears were harvested per variety. For maize flour sample preparation, kernels from the central 20 rows of each ear were sliced, immediately frozen in liquid nitrogen, and subsequently freeze‐dried for 72 hours using a vacuum freeze dryer (Gamma 1−16 LSC, CHRIST, Germany) (Supplementary Dataset 1, **Figure** **2**). The dried samples were then ground using a FW‐80 mill (Kunshan Ultrasonic Instrument Co., China), passed through a 60‐mesh sieve, and stored in sealed plastic bags at ‐20°C.

To extend the model's applicability across cereal crops, additional samples were collected, including 90 forage maize varieties, 212 rice varieties, 113 wheat varieties, and 158 barley varieties (Figure 1). Forage maize samples were harvested in 2023 from the Zhejiang A&F University farm, with 3−4 ears collected per variety. The ears were sun‐dried for 4 days, kernels removed, and then dried in a forced‐air oven at 65°C for 2 days until reaching constant weight. Samples were then ground using the FW‐80 mill. Rice grains were dehulled using an electric dehusker (Model B‐76, China), milled with a sample miller (Model JB‐20, China), and further ground into flour using a cyclone grinder (Model 3010 − 019, Fort Collins, Colorado, USA). Wheat and barley grains were sun‐dried for 4 days and directly ground into flour using the FW‐80 mill. All flour samples were passed through a 60‐mesh sieve to ensure uniform particle size (Datasets S2–S5, Supporting Information).

To simulate standard maize kernel components, synthetic samples were generated by defining the mean and variance parameters based on actual maize composition data. A total of 99 synthetic samples with diverse compositions were created using random sampling techniques. Amylose (BR, 73.6%) and protein (BR, 92.0%) were extracted from maize kernels, while sucrose (AR, 98.0%), fructose (AR, 99.0%), and glucose (AR, 99.0%) were sourced from Yuanye Bio‐Technology (Shanghai, China). Amylopectin (AR, 97.1%), also derived from maize, was obtained from Aladdin Company Ltd. (Shanghai, China), and maize crude fiber (BR, 80.0%) was provided by Zhuorui Biotechnology Co., Ltd. (Xi'an, China). The corresponding synthetic data are provided in Dataset S6 (Supporting Information), and distribution box plots are illustrated in Figure 2.

### 2.2 Spectral Acquisition

Dried flour samples of fresh maize, forage maize and barley were removed from storage and equilibrated to room temperature prior to spectral measurement. For each sample, 3 grams of flour were placed in a sample cup and analyzed using a Thermo^™^ Antaris^™^ II FT‐NIR spectrophotometer (Thermo Electron Co., USA), equipped with an interferometer and an InGaAs detector. For intact kernel samples, approximate 15 grams of fresh maize were placed in 12cm‐diameter sample cup with spinner accessory and analyzed using above same model NIR spectrophotometer. Each spectrum was recorded by averaging 32 scans. For fresh maize, spectra were collected over the range of 3999.64 to 10001.03 cm^−1^, generating 1557 data points per spectrum. For forage maize, spectra were collected from 3999.64 to 7899 cm^−1^, yielding 1012 data points per spectrum, while for barley, spectra were collected from 3999.64 to 11998.92 cm^−1^, collecting 2075 data points per spectrum. All measurements were performed in log(1/*R*) reflectance mode. Each sample was scanned twice, and the average of the two spectra was used for subsequent chemometric analysis. The laboratory temperature was maintained at approximately 25°C during all measurements. Spectral data for synthetic samples were collected using the same instrument and under the same experimental conditions as for fresh maize. Flour samples of rice and wheat were scanned using a FOSS NIRSystem 5000 spectrometer (FOSS, Denmark) in reflectance mode. Spectra were acquired over the wavelength range of 1100 − 2498 nm (equivalent to 4003.20−9090.91 cm^−1^) at 2 nm intervals, resulting in 700 data points per spectrum. All scans were also collected in log(1/*R*) mode to ensure consistency across instruments.

### 2.3 Determination of Components

The crude fiber and protein content were determined according to the Chinese national testing standards GB/T 6434‐2006, based on the media filtration method (ISO 6865:2000(E)) and GB/T 6432‐2018, derived from Kjeldahl method (ISO 8968‐1:2015), respectively. The amylopectin content was determined following the local testing standard DB32_T 2265‐2012, based on dual‐wavelength spectrophotometry.^[^ ^30^ ^]^ For sugar content, the determination of fructose, glucose, and sucrose was performed according to the National Food Safety Standard GB_5009.8‐2023, which utilizes the high performance liquid chromatography method.^[^ ^31^ ^]^

## 3 Methods of Analysis

### 3.1 Spectra Selection

The critical regions of spectra were selected according to the correlation coefficients between the spectra and components.^[^ ^5^ ^]^ In this study, we selected the spectral points with Pearson correlation coefficients greater than a specified value, referred to as the correlation coefficient threshold.

### 3.2 Principal Component Analysis (PCA)

PCA is a widely employed unsupervised linear dimension reduction technique that transforms original variables into a new set of orthogonal latent variables known as principal components (*PCs*). Each principal component (*PC*) explains a certain amount of the total information contained in the original data, with the first principal component (*PC*_1_) accounting for the largest proportion of total variance. Each subsequent principal component explains a progressively smaller portion of the remaining variance, ordered by their eigenvalue magnitudes. By removing the principal components with the lowest contribution of variance, PCA effectively reduces noise and eliminates redundant information.^[^ ^32^ ^]^

### 3.3 SELECT Algorithm

Accurate NIRS models rely heavily on the representativeness of the calibration set in reflecting the diversity of the target product. Factors such as the sample's physical condition and chemical composition can significantly influence this representativeness.^[^ ^33^ ^]^ In this study, the SELECT algorithm^[^ ^34^ ^]^ was applied to select representative samples while removing those with highly similar spectral profiles. First, spectral data were subjected to PCA to reduce dimensionality and highlight key variance. Then, a Mahalanobis distance matrix was calculated between all spectral pairs. A predefined threshold was used to define the “neighborhood” of each spectrum—any spectrum within this threshold distance from the current one was considered similar. Among such neighboring spectra, only one was retained while the others were excluded. This selection process was applied iteratively across the dataset to extract a diverse and representative subset of samples.

### 3.4 Nonlinearity Detection

To assess the nonlinearity between the spectra and components, two statistical tests were used: the Runs test and the Durbin–Watson test.^[^ ^35^, ^36^ ^]^ These tests are based on the residuals from preliminary linear regression models and identify systematic deviations that may suggest nonlinearity in relationships. Prior to conducting these tests, the component vectors were sorted accordingly.

**Runs Test**. The number of consecutive residuals with the same sign is referred to as “runs.” Since long runs are statistically unlikely, they suggest the presence of serial correlation and potentially nonlinearity. Residuals represent the difference between the observed values and the predicted values. Specifically, for a given vector $\beta ={\left(\beta_{1},\beta_{2},\text{…},\beta_{n}\right)}^{⊤}$ as the observation vector and $\hat{\beta }={\left({\hat{\beta }}_{1},{\hat{\beta }}_{2},\text{…},{\hat{\beta }}_{n}\right)}^{⊤}$ as the prediction vector, the residual vector, denoted by $e={\left(e_{1},e_{2},\text{…},e_{n}\right)}^{⊤}$, is obtained by $e=\beta -\hat{\beta }$. The number of positive residuals and negative residuals are denoted by *n*_1_ and *n*_2_, respectively. When *n*_1_ > 10 and *n*_2_ > 10, the following approximations are usually used:

(1) $$\begin{array}{cc} z & =\frac{r-\mu +0.5}{\sigma } \end{array}$$

where *r* is the number of runs, $\mu =\frac{2n_{1}n_{2}}{n_{1}+n_{2}}+1$ and $\sigma =\sqrt{\frac{2n_{1}n_{2}\left(2n_{1}n_{2}-n_{1}-n_{2}\right)}{{\left(n_{1}+n_{2}\right)}^{2}\left(n_{1}+n_{2}-1\right)}}$ are the expectation and standard deviation of the number of runs, respectively. *z* is the desired randomness measurement. The Table S1 (Supporting Information) shows that the probability of $|z|>z_{\frac{\alpha }{2}}$ is α. In other words, when |*z*| exceeds $z_{\frac{\alpha }{2}}$, the number of runs is too small, indicating a trend in residuals (i.e., the presence of nonlinearity).

**The Durbin–Watson Test**. Set the null hypothesis (*H*_0_) is no correlation in the successive residuals, and the alternative hypothesis (*H*_1_) is that the correlation exists. The Durbin–Watson test is used to evaluate *H*_0_, by testing statistic value defined by

(2) $$\begin{array}{c} d=\frac{\sum_{i=2}^{n}{\left(e_{i}-e_{i-1}\right)}^{2}}{\sum_{i=1}^{n}e_{i}^{2}}, \end{array}$$

where *e*_*i*_ is the *i*‐th entry in the residual vector. When a significance level α, sample size, and the number of regression terms (including the intercept) are given, two (lower and upper) critical values: *d*_*L*_ and *d*_*U*_, can be obtained. If *d* < *d*_*L*_, the null hypothesis is rejected, and the correlation in residuals is existed (i.e., the presence of nonlinearity). On the other hand, if *d* > *d*_*U*_, the null hypothesis can be accepted, and the correlation in residuals is negligible. If *d*_*L*_ < *d* < *d*_*U*_, the test is inconclusive.

### 3.5 PLS Method

PLS is a supervised multivariate analysis method designed to model the linear relationship between a matrix of predictor variables (X, typically spectral data) and a response matrix (Y, the dependent variables). By projecting the spectral data into latent variables, PLS maximizes the covariance between X and Y, making it especially effective for handling multicollinearity in complex datasets. The model is built by iteratively extracting components that capture the greatest shared variance between the predictor and response matrices.^[^ ^32^ ^]^

### 3.6 ANN Method

In this study, the fully‐connected feed‐forward neural network was applied. It consists of input, hidden, and output layers, where the layers are fully connected. The raw NIR spectral data are transferred into the *k* PCs by PCA, that is, *Z* = (*PC*_1_, *PC*_2_, …, *PC*_*k*_) = (*Z*_1_, *Z*_2_, …, *Z*_*n*_)^⊤^, where *k* and *n* are the numbers of PCs and samples. They are used as the inputs in the input layer with *k* neurons. With one hidden layer, the output is the estimation of each component, that is,

(3) $${\hat{y}}_{i}=\mathrm{NET}\left(Z_{i};\theta \right)=W^{2}\sigma \left(W^{1}Z_{i}+b^{1}\right)+b^{2},\;i=1,2,\text{…},n,$$

where *W* ^*l*^, *b* ^*l*^, σ(·) (*l* = 1, 2) denote the weights, bias and the activation function, respectively. θ is the set of all parameters to be optimized, including all entries of *W* ^*l*^ and *b* ^*l*^ (*l* = 1, 2). The activation functions, including *tanh*, *swish*, ReLU and Leaky_ReLU, were used in this paper. Usually, the mean squared error (MSE) between the predicted outputs ${\hat{y}}_{i}$ and the measured values *y*_*i*_, (*i* = 1, 2, ⋅⋅⋅, *n*) is adopted as the loss function, that is,

(4) $$\begin{array}{c} \min_{\theta }Loss=\frac{1}{n}\sum_{i=1}^{n}{\left(y_{i}-\mathrm{NET}\left(Z_{i};\theta \right)\right)}^{2}. \end{array}$$

The ANN model is trained by minimizing the loss function to determine the optimal parameters by Adam optimizer,^[^ ^37^ ^]^ which is denoted by $\theta^{*}$. This optimizer significantly accelerates the model's convergence through its adaptive learning rate adjustment capability, makes it suitable for optimization problems with multivariate and nonlinear characteristics, and performs well in parameter optimization tasks in the field of deep learning. With the optimal $\theta^{*}$, then the predictions of each component of the validation samples are obtained by (3), accordingly.

### 3.7 PCNN Method

Due to the limited availability of many germplasm resources, developing accurate predictive models using NIR spectra from small sample sets remains a significant challenge. To overcome this, the PCNN algorithm was introduced. This approach integrates a small number of real samples with synthetic standard samples to enhance model performance.

The PCNN framework comprises two fully connected neural networks: the PNN and CNN (Figure 1). The PNN is trained using synthetic standard samples to learn the nonlinear relationships between spectral data and component concentrations. It generates initial predictions for both calibration and validation sets. As in Equation (3), the PNN output provides the preliminary estimates for each target component.

(5) $$y_{i}^{0}={\mathrm{NET}}^{0}\left(Z_{i}^{0};\theta_{1}\right),\;i=1,2,\text{…},s,$$

where $\theta_{1}$ is the set of all parameters to be optimized, including all entries of the weights and bias in the PNN and $Z^{0}=\left(PC_{1}^{0},PC_{2}^{0},\text{…},PC_{k}^{0}\right)={\left(Z_{1}^{0},Z_{2}^{0},\text{…},Z_{s}^{0}\right)}^{⊤}$, where $PC_{i}^{0},\left(i=1,2,\text{…},k\right)$ is the *i*‐th PC vector of the spectra of synthetic standard samples after PCA, and *s* is the number of the synthetic standard samples. The loss function of PNN is defined by

(6) $$\begin{array}{c} \min_{\theta_{1}}Loss1=\frac{1}{s}\sum_{i=1}^{s}{\left({\tilde{y}}_{i}-{\mathrm{NET}}^{0}\left(Z_{i}^{0};\theta_{1}\right)\right)}^{2}, \end{array}$$

where ${\tilde{y}}_{i}\;\left(i=1,2,\text{…},s\right)$ are the measured components value of synthetic standard samples. Using PCA, the PCs vectors of the spectra of calibration and validation samples are obtained, denoted by $Z^{C}=\left(PC_{1}^{C},PC_{2}^{C},\text{…},PC_{k}^{C}\right)={\left(Z_{1}^{C},Z_{2}^{C},\text{…},Z_{p}^{C}\right)}^{⊤}$ and $Z^{V}=\left(PC_{1}^{V},PC_{2}^{V},\text{…},PC_{k}^{V}\right)={\left(Z_{1}^{V},Z_{2}^{V},\text{…},Z_{q}^{V}\right)}^{⊤}$, where *p* and *q* are the numbers of calibration and validation samples, respectively. The optimal parameter for (6) is denoted by $\theta_{1}^{*}$. Then, the prediction values of components for calibration and validation samples are obtained and denoted by ${\mathrm{NET}}^{0}\left(Z_{i}^{C};\theta_{1}^{*}\right)\;\left(i=1,2,\text{…},p\right)$ and ${\mathrm{NET}}^{0}\left(Z_{i}^{V};\theta_{1}^{*}\right)\;\left(i=1,2,\text{…},q\right)$, respectively.

The CNN is constructed similarly, and the outputs are the correction values of each component

(7) $$y_{i}^{\varepsilon }={\mathrm{NET}}^{\varepsilon }\left(Z_{i}^{C};\theta_{2}\right),\;i=1,2,\text{…},p,$$

where $\theta_{2}$ is the set of all parameters to be optimized, including all entries of the weights and bias in the CNN. The loss function of CNN is defined by

(8) $$\begin{array}{cc} \min_{\theta_{2}}\mathrm{Loss}2 & =\frac{1}{p}\sum_{i=1}^{p}{\left(y_{i}^{C}-{\mathrm{NET}}^{0}\left(Z_{i}^{C};\theta_{1}^{*}\right)-{\mathrm{NET}}^{\varepsilon }\left(Z_{i}^{C};\theta_{2}\right)\right)}^{2}, \end{array}$$

where $y_{i}^{C}\;\left(i=1,2,\text{…},p\right)$ are the measured component values of calibration samples. The optimal parameter for (8) is denoted by $\theta_{2}^{*}$. Then, the correction values of each component for calibration and validation samples are obtained and denoted by ${\mathrm{NET}}^{\varepsilon }\left(Z_{i}^{C};\theta_{2}^{*}\right)\;\left(i=1,2,\text{…},p\right)$ and ${\mathrm{NET}}^{\varepsilon }\left(Z_{i}^{V};\theta_{2}^{*}\right)\;\left(i=1,2,\text{…},q\right)$, respectively. Finally, the total estimations of each component for calibration and validation samples are obtained by

(9) $$\begin{array}{cc} {\hat{y}}_{i}^{C} & ={\mathrm{NET}}^{0}\left(Z_{i}^{C};\theta_{1}^{*}\right)+{\mathrm{NET}}^{\varepsilon }\left(Z_{i}^{C};\theta_{2}^{*}\right),\;i=1,2,\text{…},p, \end{array}$$

(10) $$\begin{array}{cc} {\hat{y}}_{i}^{V} & ={\mathrm{NET}}^{0}\left(Z_{i}^{V};\theta_{1}^{*}\right)+{\mathrm{NET}}^{\varepsilon }\left(Z_{i}^{V};\theta_{2}^{*}\right),\;i=1,2,\text{…},q, \end{array}$$

respectively.

### 3.8 Model Evaluation

In this study, the samples were divided into two subsets: the calibration set and the validation set. The calibration set was used for training the model, while the validation set was used to evaluate the model's performance and robustness. The prediction accuracy of the models is assessed using the coefficient of determination, denoted by *R* ^2^, and the root mean square error (RMSE), defined as follows:

(11) $$\begin{array}{cc} R^{2} & =1-\frac{\sum_{i=1}^{n}{\left(y_{i}-{\hat{y}}_{i}\right)}^{2}}{\sum_{i=1}^{n}{\left(y_{i}-\bar{y}\right)}^{2}}, \end{array}$$

(12) $$\begin{array}{cc} \text{RMSE} & =\sqrt{\frac{\sum_{i=1}^{n}{\left(y_{i}-{\hat{y}}_{i}\right)}^{2}}{n}}, \end{array}$$

where *n* is the number of samples and ${\hat{y}}_{i}$, *y*_*i*_, and $\bar{y}$ are the prediction, measurements, and mean value of measurements, respectively. For the calibration and the validation samples, the determination coefficient and the root mean square errors are calculated and denoted by $R_{C}^{2}$, $R_{V}^{2}$, RMSEC and RMSEV, respectively. The value of RPD is calculated by taking the ratio of the standard deviation of the validation set, denoted by *S*, to RMSEV. That is,

(13) $$\begin{array}{cc} S & =\sqrt{\frac{\sum_{i=1}^{q}{\left(y_{i}-\bar{y}\right)}^{2}}{q-1}}, \end{array}$$

(14) $$\begin{array}{cc} \text{RPD} & =\frac{S}{\text{RMSEV}}, \end{array}$$

where *q* is the number of validation samples and *y*_*i*_, and $\bar{y}$ are the measurements and mean value of measurements of validation sample, respectively. The RPD value is a estimator to standardize predictive accuracy and check the robustness of a calibration model. The higher the RPD value is, the better capability of prediction is.

### 3.9 Statistical Analysis

Statistical analyses were conducted on a system equipped with an Intel Core i5‐13500 CPU and 32 GB of RAM. Nonlinear relationships between spectra and components were assessed using D‐W and Runs tests. Sample selection was performed using the SELECT algorithm, and spectral feature selection was based on Pearson correlation coefficients; these analyses were implemented in MATLAB R2018b. PCA dimensionality reduction and PLS regression were implemented in both MATLAB R2018b and Python. Component contents were normalized using min‐max scaling during preprocessing in Python. ANN and PCNN models were developed in Python using the PyCharm IDE. The implementation leveraged libraries including NumPy, Pandas, and Scikit‐learn, with neural network architectures constructed using TensorFlow 2.6.0.

## 4 Results

### 4.1 Nonlinear Detection for NIR Modeling

**Figure 3:**
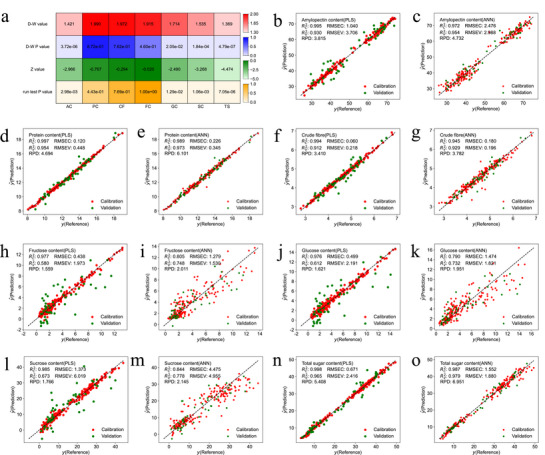
Nonlinear detection, calibration and validation effects of PLS and ANN methods with abundant calibration samples for seven components. Nonlinear detection heatmap for NIR modeling of amylopectin (AC), protein (PC), crude fiber (CF), fructose (FC), glucose (GC), sucrose (SC) and total sugar (TS) (a), calibration and validation effects of PLS and ANN methods for amylopectin, protein, crude fiber, fructose, glucose, sucrose and total sugar with 195 calibration samples (represented by red dots) and 51 validation samples (represented by green dots) (b–o).

Nonlinear detections of seven kernel components – amylopectin (AC), protein (PC), crude fiber (CF), fructose (FC), glucose (GC), sucrose (SC), and total sugar (TS) – was conducted using the D‐W and runs tests (**Figure** **3**a). The analysis showed that AC, GC, SC, and TS exhibited nonlinear relationships with the spectral data, while PC, CF, and FC showed linear relationships.

Specifically, for AC, GC, SC, and TS, the D‐W values were below the lower critical value (*d*_*L*_ = 1.738), and the absolute z‐values exceeded 2.490 (|*z*| > *z*_0.025_ = 1.960), with p‐values <0.05. These results led to rejection of the null hypothesis (*H*_0_), confirming nonlinearity. In contrast, PC, CF, and FC had D‐W values above the upper critical value (*d*_*U*_ = 1.799), absolute z‐values below 0.767 (|*z*| < *z*_0.025_ = 1.960), and p‐values ⩾0.443, indicating no significant deviation from linearity and supporting the assumption of a linear relationship with the spectra.

### 4.2 PLS and ANN Methods With Abundant Calibration Samples

Using random sampling, the 246 samples were split into a calibration set of 195 samples and a validation set of 51 samples. The PLS calibration models for the seven components – amylopectin, protein, crude fiber, fructose, glucose, sucrose, and total sugar – achieved RPD values of 3.815, 4.694, 3.410, 1.559, 1.621, 1.766, and 5.408, respectively. Corresponding coefficients of determination ($R_{V}^{2}$) for the validation set were 0.930, 0.954, 0.912, 0.580, 0.612, 0.673, and 0.965, respectively (Figure 3b,d,f,h,j,l,n). These results indicate strong predictive performance for amylopectin, protein, crude fiber, and total sugar, all with RPD values above 3 and $R_{V}^{2}$ values exceeding 0.912. In contrast, models for fructose, glucose, and sucrose showed weaker performance, with RPD values below 2 and $R_{V}^{2}$ values under 0.673.

For the ANN calibration models, RPD values improved to 4.732, 6.101, 3.782, 2.011, 1.951, 2.145, and 6.951, respectively, alongside $R_{V}^{2}$ values of 0.954, 0.973, 0.929, 0.748, 0.732, 0.778, and 0.979 (Figure 3c,e,g,i,k,m,o). This demonstrates strong predictive accuracy for amylopectin, protein, crude fiber, and total sugar (RPD >3, $R_{V}^{2}\geq 0.929$), as well as improved predictions for fructose, glucose, and sucrose (RPD ⩾1.951, $R_{V}^{2}\geq 0.732$). Overall, the ANN models exhibited superior robustness and accuracy compared to the PLS models.

Based on previous nonlinear analysis, protein, crude fiber, and fructose showed linear relationships with the spectra, so the ANN models for these components employed the ReLU activation function. Conversely, amylopectin, glucose, sucrose, and total sugar exhibited nonlinear spectral relationships, prompting the use of *tanh* and *swish* activation functions for their respective ANN models (**Table** **1**). This adaptability enabled the ANN to effectively analyze both high‐dimensional linear and nonlinear data, extracting deeper spectral features for improved predictions.

**Table 1:** The optimal parameters of ANN method for seven components.

Components	Number of neurons	Activation function	Epochs
Amylopectin content	28	*tanh*	4000
Protein content	9	ReLU	4000
Crude fiber	1	ReLU	600
Fructose content	6	ReLU	1000
Glucose content	6	*swish*	1700
Sucrose content	5	*tanh*	1000
Total sugar content	4	*tanh*	5500

### 4.3 PCNN Method With Small Calibration Samples

Due to the high cost, time‐consuming nature of wet chemistry analyses, and limited germplasm variability in calibration samples, we aimed to develop robust and accurate calibration models using a small number of real samples supplemented with expanded synthetic spectral data. To achieve this, a PCNN method was specifically designed for small sample scenarios. The approach incorporates several optimization strategies, including spectral fragment selection, PC number tuning, NH distance thresholds, and neural network parameter adjustments.

#### 4.3.1 Optimizations of Modeling Parameters

To evaluate the robustness of the PCNN method, ten calibration sets were generated using the SELECT algorithm, each derived after a random shuffle of the raw spectral data. Model performance was assessed using three indicators – RPD, $R_{V}^{2}$, and RMSEV – with results averaged across the tests.

**Figure 4:**
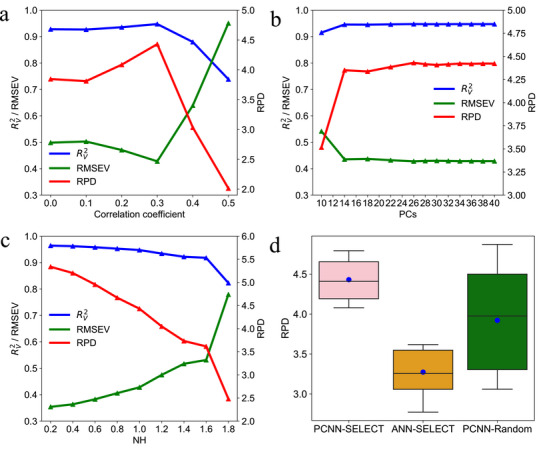
Optimizations of modeling parameters for PCNN method. Variation of correlation coefficient from 0 to 0.5, the line chart of $R_{V}^{2}$, RMSEV and RPD were plotted (a). The left axis represents the value of $R_{V}^{2}$ and RMSEV, and the right axis represents the RPD value. Variation of PCs from 10 to 40 (b), and NH distances, setting by 0.2 (180 calibration samples), 0.4 (123 calibration samples), 0.6 (77 calibration samples), 0.8 (48 calibration samples), 1.0 (32 calibration samples), 1.2 (22 calibration samples), 1.4 (17 calibration samples), 1.6 (13 calibration samples), 1.8 (9 calibration samples) (c), the line chart of $R_{V}^{2}$, RMSEV and RPD were plotted. Box plot of RPD was shown for PCNN‐SELECT, ANN‐SELECT and PCNN‐Random (d).

A correlation analysis between spectral variation and component concentrations was conducted to guide spectral filtering. Threshold values of 0, 0.1, 0.2, 0.3, 0.4, and 0.5 were applied, where a threshold of 0 represented use of the full spectrum. Protein prediction was optimized by identifying the most effective threshold. As the correlation threshold increased, the values of RPD, $R_{V}^{2}$, and RMSEV varied. At a threshold of 0.3, both RPD and $R_{V}^{2}$ reached their peak, while RMSEV was minimized, indicating this as the optimal threshold for protein calibration (**Figure** **4**a). It was also noted that selecting too few wavelength points failed to capture sufficient spectral information. Therefore, a correlation threshold of 0.3 was similarly applied to identify relevant spectral fragments for predicting amylopectin, crude fiber, and total sugar. Compared to using the full spectrum, spectral selection at this level improved the overall prediction accuracy (**Table** **2**).

**Table 2:** Comparison of model performance using full spectra and spectra selection for four components.

Components	Evaluation indicators	Full spectrum	Spectra selection
Amylopectin content	$R_{C}^{2}$	0.984	0.981
	$R_{V}^{2}$	0.892	0.922
	RMSEC	1.703	1.798
	RMSEV	4.548	3.839
	RPD	3.087	3.716
Protein content	$R_{C}^{2}$	0.996	0.986
	$R_{V}^{2}$	0.928	0.948
	RMSEC	0.138	0.246
	RMSEV	0.499	0.428
	RPD	3.845	4.431
Crude fiber	$R_{C}^{2}$	0.985	0.971
	$R_{V}^{2}$	0.837	0.869
	RMSEC	0.089	0.126
	RMSEV	0.267	0.240
	RPD	2.549	2.821
Total sugar content	$R_{C}^{2}$	0.998	0.992
	$R_{V}^{2}$	0.914	0.951
	RMSEC	0.434	1.016
	RMSEV	3.430	2.620
	RPD	3.764	4.862

Subsequently, the effect of varying the number of PCs on protein prediction was evaluated using the PCNN method, with the number of PCs ranging from 10 to 40. Results showed that increasing the PCs from 10 to 12 led to a significant decrease in the $R_{V}^{2}$ value. Both the RPD and $R_{V}^{2}$ values rose sharply, reaching their peak, while the RMSEV reached its lowest value at 26 PCs. Beyond 26 PCs, from 26 to 40, all three evaluation metrics stabilized, indicating that 26 PCs are sufficient for accurate quantitative analysis (Figure 4b).

The influence of the NH distance parameter, which guides calibration set selection via the SELECT algorithm, was also investigated. Set the NH distance by 0.2 (180 calibration samples), 0.4 (123 calibration samples), 0.6 (77 calibration samples), 0.8 (48 calibration samples), 1.0 (32 calibration samples), 1.2 (22 calibration samples), 1.4 (17 calibration samples), 1.6 (13 calibration samples), 1.8 (9 calibration samples) (Table S3, Supporting Information), respectively. As the NH distance increased from 0.2 to 1.0, both RPD and $R_{V}^{2}$ values gradually declined, while RMSEV increased, reflecting a decrease in calibration sample size. When the NH distance exceeded 1.0, there was a sharp drop in RPD and $R_{V}^{2}$ values. Therefore, an NH distance cutoff of 1.0 was deemed optimal for protein prediction, balancing sample reduction with robust predictive performance (Figure 4c).

Using 10 calibration sets, each averaging 32 samples selected either by the SELECT algorithm (NH distance = 1.0) or by random sampling, three strategies were compared: PCNN with SELECT, PCNN with Random sampling, and ANN with SELECT. The distribution of RPD values for these methods was illustrated via box plots. The results demonstrated that PCNN combined with the SELECT algorithm outperformed ANN with SELECT. Moreover, PCNN's predictive accuracy was higher when using SELECT‐selected calibration sets compared to random sampling. The average RPD values for PCNN‐SELECT, ANN‐SELECT, and PCNN‐Random were 4.431, 3.273, and 3.922, respectively (Figure 4d).

#### 4.3.2 Components Evaluation Based on PCNN

The PCNN model was constructed with specified numbers of neurons, activation functions, and epochs in both the PNN and CNN layers for amylopectin, protein, crude fiber, and total sugar (**Table** **3**), as well as for fructose, glucose, sucrose, and total sugar (**Table** **4**).

**Table 3:** The optimal parameters of PCNN method for amylopectin, protein, crude fiber, and total sugar.

	Prediction neural network			Correction neural network		
Components	Number of neurons	Activation function	Epochs	Number of neurons	Activation function	Epochs
Amylopectin content	9	Leaky_ReLU	1100	4	*swish*	3200
Protein content	10	*tanh*	300	2	*tanh*	3000
Crude fiber	10	*tanh*	100	18	*tanh*	800
Total sugar content	1	*tanh*	3300	36	*tanh*	3200

**Table 4:** The optimal parameters of PCNN method for fructose, glucose, sucrose, and total sugar.

	Prediction neural network			Correction neural network		
Components	Number of neurons	Activation function	Epochs	Number of neurons	Activation function	Epochs
Fructose content	1	*tanh*	600	2	*swish*	3000
Glucose content	10	Leaky_ReLU	150	10	*swish*	1000
Sucrose fiber	31	ReLU	900	4	*tanh*	1900
total sugar content	10	Leaky_ReLU	100	10	Leaky_ReLU	1000

**Figure 5:**
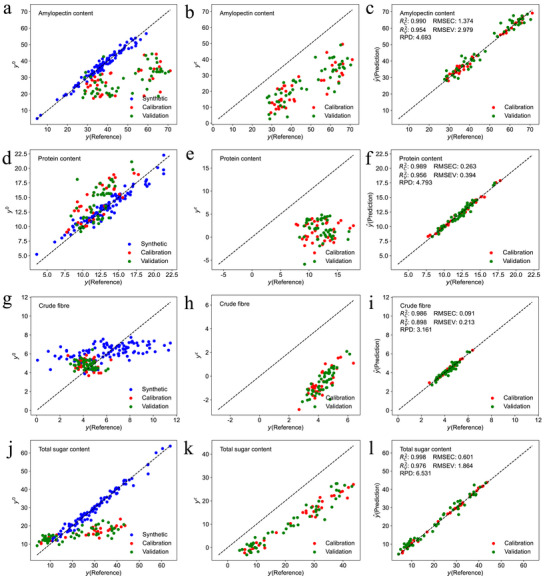
Calibration and validation effects of PCNN method with small calibration samples for amylopectin, protein, crude fiber and total sugar with PCNN method. Using the calibration set 2 (Table S2, Supporting Information) to estimate amylopectin content, the prediction value (*y* ^0^) of PNN vs the reference (a), the correction value (*y* ^ε^) of CNN vs the reference (b) and the total estimation value ($\hat{y}$) vs reference (c) were illustrated. Similarly, using the calibration sets 1, 7 and 9 to estimate the contents of protein, crude fiber and total sugar, respectively, the prediction values of PNN vs the reference (d, g, j), the correction values of CNN vs the reference (e, h, k) and the total estimation values vs reference (f, i, l) were illustrated. The blue, red and green dots represent the predictive effects of the synthetic samples, calibration samples and validation samples, respectively.

For amylopectin, protein, crude fiber, and total sugar, the optimal calibration sets – sets 2, 1, 7, and 9 containing 35, 34, 30, and 31 samples, respectively – were selected for demonstration (Table S2, Supporting Information; **Figure** **5**). In the PNN, trained on 99 synthetic samples, predictions for amylopectin and total sugar tended to underestimate actual values, whereas protein and crude fiber predictions were overestimated (Figure 5a,d,g,j). Moreover, it reflected the greater diversity of synthetic samples compared to the real maize data, as the synthetic spectra covered a wider range. Subsequently, the CNN adjusted these positive and negative deviations using a small number of calibration samples (Figure 5b,e,h,k). The combined outputs from PNN and CNN achieved RPD values of 4.693, 4.793, 3.161, and 6.531; $R_{V}^{2}$ values of 0.954, 0.956, 0.898, and 0.976; and RMSEV values of 2.979, 0.394, 0.213, and 1.864 for amylopectin, protein, crude fiber, and total sugar, respectively (Figure 5c,f,i,l). These high RPD and $R_{V}^{2}$ values indicate strong predictive accuracy and robust generalization of the PCNN method for these components.

**Figure 6:**
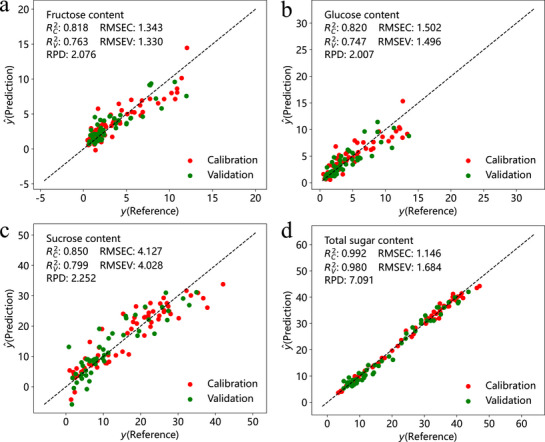
Calibration and validation effects of PCNN method with small calibration samples for fructose, glucose, sucrose and total sugar. The prediction values $\hat{y}$ of PCNN vs reference *y* for fructose (a), glucose (b), sucrose, (c) and total sugar (d) were illustrated. The red and green dots represent the predictive effects of the 62 calibration samples and 51 validation samples, respectively.

To enhance prediction accuracy for fructose, glucose, and sucrose, 62 calibration samples were selected using the SELECT algorithm with an NH distance threshold above 0.7. Additionally, 51 validation samples with NH distances below 0.26 were used to assess model performance. The PCNN method yielded RPD values of 2.076, 2.007, 2.252, and 7.091, and $R_{V}^{2}$ values of 0.763, 0.747, 0.799, and 0.980 for fructose, glucose, sucrose, and total sugar, respectively (**Figure** **6**a–d). In summary, effective calibration models for all sugar components were successfully developed using relatively small sample sizes.

### 4.4 Scalability of PCNN

**Figure 7:**
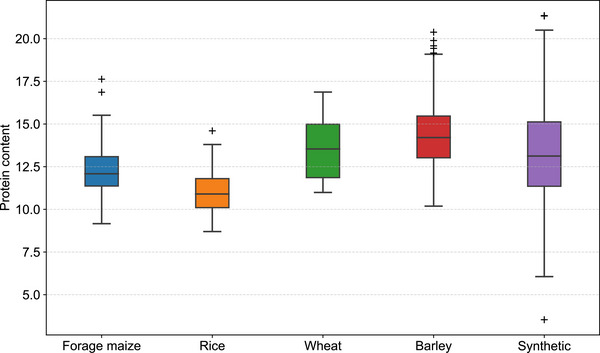
Box plots of protein content distributions in forage maize, rice, wheat, barley, and synthetic standard samples.

To assess the applicability of the proposed PCNN method beyond fresh maize, flour samples from mature forage maize (90 varieties), rice (212 varieties), wheat (113 varieties), and barley (158 varieties) were collected for protein content determination and NIR spectral scanning, and the box plots of protein distributions in four cereal samples and synthetic standard samples were illustrated in **Figure** **7**. For each cereal, 30 calibration samples were selected using the SELECT algorithm with NH distance thresholds set at 0.65, 1.144, 0.68, and 1.15, respectively. The remaining samples – 60 forage maize samples, 182 rice samples, 83 wheat samples, and 128 barley samples – served as validation sets. Optimal parameters for the ANN and PCNN models for these cereals were listed in Tables S4 and S5 (Supporting Information), respectively.

**Figure 8:**
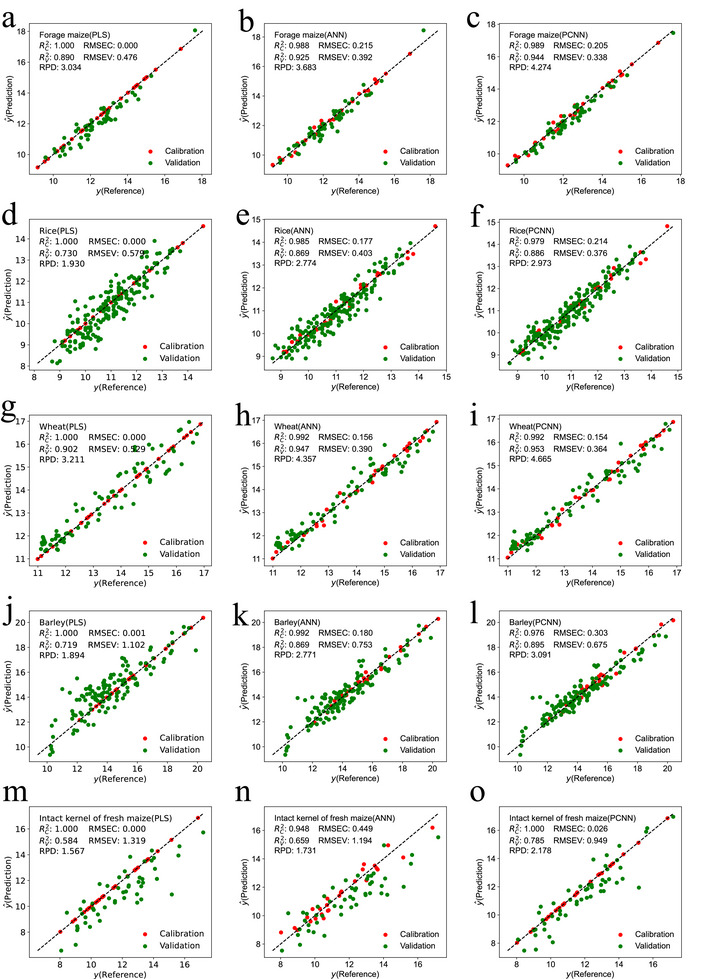
Comparison of calibration and validation effects of cereals with PLS, ANN and PCNN method. The data of forage maize, rice, wheat and barley with 90, 212, 113, 158 samples were collected to validate the scalability of PCNN. And the data of 29 calibration samples and 51 validation samples were used to validate the PCNN models of intact fresh maize kernels. The prediction values $\hat{y}$ of PLS (a, d, g, j, m), ANN (b, e, h, k, n), PCNN (c, f, i, l, o) versus reference *y* for protein were illustrated. The red and green dots represent the predictive effects of the calibration samples and validation samples, respectively.

Compared to PLS and ANN, the PCNN method showed significant improvements in protein prediction performance. For forage maize, PCNN increased RPD values by 40.87% (1.240/3.034) and 16.05% (0.591/3.683), and improved $R_{V}^{2}$ values by 6.07% (0.054/0.890) and 2.05% (0.019/0.925), respectively. RMSEV decreased by 28.99% (0.138/0.476) and 13.78% (0.054/0.392) (**Figure** **8**a–c). For rice protein prediction, PCNN improved RPD values by 54.04% (1.043/1.930) and 7.17% (0.199/2.774), increased $R_{V}^{2}$ values by 21.37% (0.156/0.730) and 1.96% (0.017/0.869), and reduced RMSEV by 35.06% (0.203/0.579), 6.70% (0.027/0.403) (Figure 8d–f). For wheat, the PCNN method yielded RPD improvements of 45.28% (1.454/3.211) and 7.07% (0.308/4.357), $R_{V}^{2}$ increases of 5.65% (0.051/0.902) and 0.63% (0.006/0.947), and RMSEV reductions of 31.19% (0.165/0.529), 6.67% (0.026/0.390) (Figures 8g–i). In barley, PCNN achieved RPD gains of 63.20% (1.197/1.894) and 11.55% (0.320/2.771), $R_{V}^{2}$ improvements of 24.48% (0.176/0.719) and 2.99% (0.026/0.869), and RMSEV decreases of 38.75% (0.427/1.102) and 10.36% (0.078/0.753) (Figure 8j–l).

To validate the performance of the developed PCNN models on intact kernels, the 246 intact kernels samples of fresh maize were collected (Dataset S7, Supporting Information). For these samples, 51 validation samples were selected using the SELECT algorithm with NH distance thresholds set at 0.2625, then 29 calibration samples were selected with NH distance thresholds equals 1.0. Compared the predictive effects with PLS and ANN, PCNN increased RPD values by 38.99% (0.611/1.567) and 25.82% (0.447/1.731), and improved $R_{V}^{2}$ values by 34.42% (0.201/0.584) and 19.12% (0.126/0.659), respectively. RMSEV decreased by 28.05% (0.37/1.319) and 20.52% (0.245/1.194) (Figure 8m–o).

These results demonstrate that the PCNN method is a robust, generalizable, and scalable approach for modeling the relationship between NIR spectra and component content across multiple cereal grains. By leveraging initial predictions from PNN and refining them with a small calibration set in CNN, the method effectively handles spectra from different NIR spectrometers. This approach captures complex dataset relationships, exhibiting strong generalization across diverse maize varieties, sample types, and cereal species.

## 5 Discussion

### 5.1 Sampling and Spectra Selection

Sample selection for calibration and validation plays a critical role in determining the predictive accuracy of NIR models. Traditional approaches, such as the Kennard–Stone (KS) algorithm,^[^ ^38^ ^]^ are widely used in machine learning and spectral analysis to ensure diversity among selected samples by maximizing their spatial separation. In this study, the SELECT algorithm^[^ ^34^ ^]^ was applied to identify a representative subset of samples. This method calculates Mahalanobis distances between the PCs of sample spectra, ensuring broad coverage across the spectral feature space. Compared to random sampling, the PCNN model built using SELECT‐selected samples demonstrated notably improved predictive performance.

To comprehensively evaluate the performance of the PCNN model, optimize key parameters (including correlation coefficient threshold, NH distance, number of principal components, and neural network settings), and reduce potential bias from a single dataset, the raw sample set was randomly shuffled 10 times. For each shuffle, the SELECT algorithm was applied to generate averaging 32 samples. In parallel, 10 randomly selected sample sets (also with 32 samples each) were created to serve as a comparative baseline. This strategy enabled a robust assessment of the PCNN model's stability, generalization, and predictive reliability. While 32 samples were sufficient for accurate prediction of amylopectin, protein, and crude fiber, the calibration sample size was increased to 62 for fructose, glucose, and sucrose (Figure 6). This increase was necessary due to the similar chemical structures of these sugars, which produce overlapping NIR spectral features and limit the discrimination ability of individual models. Furthermore, the relatively low concentrations of glucose and fructose reduced calibration accuracy (Figure 3). In contrast, the total sugar model, which captures the integrated spectral features of all sugars, demonstrated improved robustness and predictive accuracy by minimizing signal interference (Figures 3 and 5).

It is well established that the dominant absorption bands in the NIR region primarily arise from overtone and combination vibrations of hydrogen‐containing functional groups in organic compounds.^[^ ^39^, ^40^, ^41^ ^]^ As a result, selecting the most informative spectral fragments is essential for building accurate and efficient models. In this study, correlation coefficients between spectral data and component concentrations were calculated to guide fragment selection. A threshold of 0.3 was identified as optimal, with all four target components demonstrating improved model performance using the selected spectral regions compared to the full spectra. This observation is consistent with previous findings in studies on flavonoid composition prediction in okra pods.^[^ ^5^ ^]^

### 5.2 PCNN: Nonlinearity, Small Samples, and Scalability

The absorbance behavior of NIR spectra in different maize samples is inherently nonlinear. ANN is particularly effective in capturing this complexity by modeling nonlinear relationships between NIR spectral inputs and chemical component outputs.^[^ ^21^, ^42^, ^43^ ^]^ As shown in Figure 3, the ANN method consistently outperforms PLS regression in predictive accuracy‐whether for components that follow relatively linear trends, such as protein, crude fiber, and fructose, or those exhibiting more nonlinear patterns, like amylopectin, glucose, sucrose, and total sugar.

However, a sufficient amount of measurement data is necessary to establish a satisfactory nonlinear model using the ANN method. The previous research extensively studied the effect of sample size on model performance and concluded that the model's performance improves as the number of samples in the calibration set increases.^[^ ^25^ ^]^ In real‐world situations, datasets are often limited, unbalanced, or require substantial resources to collect large amounts of sample data. To address these challenges and enhance prediction accuracy, a novel method called PCNN was proposed. As shown in Figure 5, with 35, 34, 30, and 31 validation samples, respectively, the PCNN method demonstrates high accuracy in predicting components like amylopectin, protein, crude fiber, and total sugar, with RPD values of 4.693, 4.793, 3.161, and 6.531, and $R_{V}^{2}$ values of 0.954, 0.956, 0.898, and 0.976.

Unlike synthetic data generated by deep convolutional generative adversarial networks^[^ ^25^ ^]^ – which often fail to enhance the generalization ability of regression models due to their focus on central cluster characteristics – the 99 synthetic samples used in this study exhibited greater diversity. This variability provided valuable training input for the PNN, improving the initial predictions of component values. These preliminary estimates were then refined using a small set of real maize samples through the CNN, significantly enhancing overall prediction accuracy.

The PCNN approach was also successfully extended to predict protein content in other cereals, including forage maize, rice, wheat, and barley. In these cases, the same synthetic samples trained the PNN, while 30 real cereal samples formed the CNN calibration set. Validation was performed on the remaining 60 (forage maize), 182 (rice), 83 (wheat), and 128 (barley) samples. Compared to PLS and ANN models, the PCNN showed notable improvements in RPD – by 40.87% and 16.05% (forage maize), 54.04% and 7.17% (rice), 45.28% and 7.07% (wheat), and 63.20% and 11.55% (barley). $R_{V}^{2}$ values also increased by 6.07% and 2.05%, 21.37% and 1.96%, 5.65% and 0.63%, and 24.48% and 2.99%, respectively (Figure 8). These results highlight the robustness and adaptability of the PCNN method, demonstrating its effectiveness even with limited calibration data and its potential for broader application across diverse cereal types using NIR spectroscopy.

### 5.3 Improvement and Application

There are two main areas for improvement in future research. First, sample size remains a critical factor affecting both experimental efficiency and cost. Advancing modeling algorithms and feature extraction techniques to develop predictive models that require fewer calibration samples without compromising accuracy will be essential. Second, this study relied on freeze‐dried maize kernel powder, which, while effective, limits practical applicability. Using intact maize kernels or whole cobs for spectral data collection would offer a more convenient and time‐efficient alternative. Though with small number of intact fresh kernels as calibration set (NH = 1.0, and 29 intact samples), PCNN model is superior compared to the PLS and ANN models, compared with the results using the powder samples (see Figures 8o and 5f), the RPD value and $R_{V}^{2}$ value using intact kernels are far inferior to those using powder kernels. These enhancements would improve scalability and practicality, making the method more suitable for real‐world applications and further research.

Furthermore, for plant types with limited availability – such as rare crops, specialty vegetables, or traditional Chinese medicinal plants – gathering a sufficiently large and diverse set of samples poses significant challenges, making it difficult to develop reliable NIR calibration models. These situations typically involve two key issues: limited compositional variability and a small number of available samples. To address the first challenge, a random blending approach was introduced, combining standard samples based on key components to generate synthetic data with expanded variation. To overcome the second, we designed a streamlined analytical model that reduces the number of calibration samples needed by incorporating wavelength selection, NH distance tuning, and optimization of the PCNN activation functions. As a result, the proposed PCNN approach presents a practical and effective solution for building NIR calibration models in plant products where sample resources are limited.

**Figure 9:**
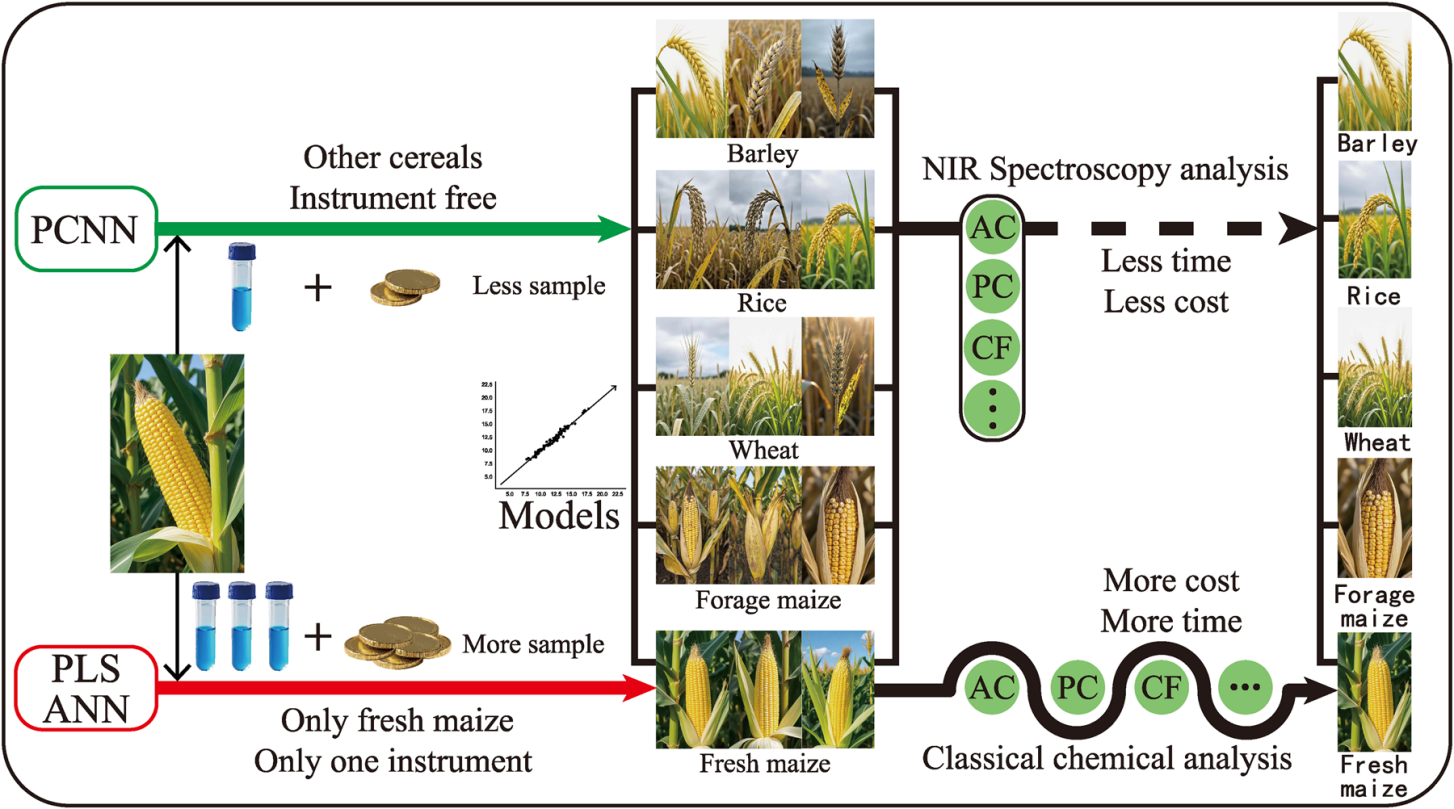
The schematic diagram demonstrates the time and cost‐saving, compatible of different NIR spectrometers and extensible PCNN method for breeding application.

The proposed PCNN method offers significant savings in both time and cost. Traditional chemical analysis of the seven target components across 246 fresh maize samples would require approximately 338.25 working days (assuming 8 hours per day) and cost around 150,000 RMB. In contrast, conventional PLS and standard ANN models demand large sample sizes to achieve high accuracy, resulting in time‐consuming, labor‐intensive, and expensive workflows. The PCNN approach, however, requires only about 30 real samples – when combined with synthetic data – to build an accurate calibration model, reducing time and financial costs by at least two‐thirds. Moreover, the PCNN method demonstrates strong scalability. It has been successfully adapted for protein prediction in other cereals such as forage maize, rice, wheat, and barley, even when spectra were obtained using different NIR instruments (**Figure** **9**). This highlights the method's potential for broader application, particularly for compositional analysis of plant products with limited sample availability. Once NIR calibration models for the seven components are established, future measurements can be completed in one minute, with negligible ongoing cost – limited to instrument depreciation. Thus, as a rapid, nondestructive, and cost‐efficient tool for evaluating compositional traits, the PCNN method is well suited for plant breeding applications, helping to accelerate selection and improve breeding program efficiency.

## 6 Conclusion

Developing accurate NIR calibration models with limited sample sizes remains a major challenge. To address this, the PCNN method was proposed, integrating the SELECT algorithm for optimal sample selection and incorporating multiple parameter optimization strategies – such as tuning NH distances, the number of principal components (PCs) and neurons, activation functions, and spectral regions. The results demonstrate that PCNN delivers high prediction accuracy across various components, enabling comprehensive and reliable quality evaluation of fresh maize. Moreover, the PCNN approach was successfully extended to predict protein content in forage maize, rice, wheat, and barley, achieving performance comparable to that of more sample‐intensive methods. These findings underscore the potential of PCNN as a powerful, scalable solution for compositional analysis of maize and other cereal grains. As a rapid, cost‐competitive, and non‐destructive method, PCNN is particularly well suited for quality trait selection in cereal breeding programs.

## Conflict of Interest

The authors declare no conflict of interest.

## Author Contributions

J.S., E.Y., and X.Z. contributed equally to this work. J.S. contributed to writing (review and editing), supervision, investigation, and conceptualization. E.Y. was responsible for writing the original draft, validation, methodology, investigation, and formal analysis. X.Z. contributed to writing the original draft, validation, software development, visualization, and data curation. L.Z. participated in validation, funding acquisition, and investigation. W.C. contributed to software, methodology, funding acquisition, conceptualization, and validation. X.Y. was involved in validation, methodology, and formal analysis. H.H. contributed to visualization, software, and data curation. Y.Q. worked on methodology and validation. Z.Z. contributed to writing (review and editing), supervision, methodology, conceptualization, funding acquisition, and formal analysis. J.W. contributed to writing (review and editing), validation, supervision, investigation, methodology, and conceptualization.

## Supporting information

Supporting Information

## Data Availability

The data that support the findings of this study are available in the supplementary material of this article.
