# Supplementary material for: A Cost‐Effective and Scalable Machine Learning Approach for Quality Assessment of Fresh Maize Kernel Using NIR Spectroscopy
Source: Adv Sci (Weinh). 2025 Sep 29;12(46):e12750. doi: 10.1002/advs.202512750 (PMC12697840; doi:10.1002/advs.202512750)
Supplement: Supplementary file 1 — Supporting Information [file ADVS-12-e12750-s001.pdf]

Appendix

Table S1: Critical values for runs test.

α	0.400	0.300	0.200	0.100	0.050	0.025	0.020	0.010	0.005	0.001
$z_{\frac{\alpha}{2}}$	0.842	1.036	1.282	1.654	1.960	2.240	2.326	2.576	2.807	3.291

Table S2: 10 calibration sets with NH=1.0.

set1	set2	set3	set4	set5	set6	set7	set8	set9	set10
1	3	1	20	3	3	1	14	3	3
2	8	14	36	5	5	7	32	6	5
3	31	20	39	8	8	20	36	8	17
4	41	41	41	25	16	27	49	11	20
5	46	42	43	33	18	32	52	12	40
6	50	43	62	40	23	41	78	35	43
7	53	53	63	52	44	53	89	41	50
8	63	58	70	53	46	59	91	43	59
11	70	66	73	59	51	63	94	46	62
12	73	70	74	62	54	70	95	53	64
21	90	78	76	70	65	71	108	54	65
23	97	93	80	79	77	80	110	70	73
28	103	95	88	90	92	84	112	71	80
41	110	97	95	96	97	94	121	83	97
42	111	111	97	97	105	111	129	90	110
43	112	121	105	105	106	113	133	97	111
45	113	123	109	108	107	121	134	107	112
53	115	125	121	111	109	127	137	111	119
56	121	129	122	119	110	128	138	112	121
59	129	133	124	121	111	129	140	113	125
66	131	138	129	124	121	133	143	115	130
83	133	139	133	127	123	138	156	121	131
90	134	148	134	129	125	142	174	124	148
94	137	153	135	133	139	155	181	131	154
97	140	163	138	138	144	156	183	139	161
121	143	166	148	144	151	164	184	153	163
138	145	178	153	151	160	181	188	179	179
140	156	182	156	171	180	184	193	184	180
148	169	184	167	172	184	190		189	184
160	172	188	172	178	188	191		190	188
182	181	190	173	184	189			195	189
184	184	192	184	185	190				190
188	188		189	188					
190	189		193	190					
	190								

Table S3: Sample numbers of 10 calibration sets with different NH values.

Sets	NH values								
	0.2	0.4	0.6	0.8	1.0	1.2	1.4	1.6	1.8
set1	180	126	80	49	34	20	18	13	10
set2	180	123	79	46	35	22	15	12	9
set3	180	123	78	47	32	21	19	12	9
set4	179	124	78	48	34	21	16	15	10
set5	180	122	74	51	34	22	19	14	9
set6	179	123	74	50	32	21	18	12	10
set7	179	124	77	49	30	23	15	12	8
set8	180	124	75	48	28	23	17	12	10
set9	180	119	76	46	31	22	17	11	10
set10	179	124	79	46	32	20	18	12	9
average numbers	180	123	77	48	32	22	17	13	9

Table S4: The optimal parameters of ANN method for different cereals.

Cereals	Number of neurons	Activation function	Epochs
Forage maize	7	relu	2600
Rice	11	relu	3000
Wheat	28	tanh	2700
Barley	38	tanh	4000
Fresh maize (intact kernels)	5	tanh	2000

Table S5: The optimal parameters of PCNN method for different cereals.

Cereals	Prediction neural network			Correction neural network		
	Number of neurons	Activation function	Epochs	Number of neurons	Activation function	Epochs
Forage maize	1	*tanh*	1500	17	*tanh*	2600
Rice	13	*relu*	2100	49	*relu*	2800
Wheat	6	*swish*	3000	45	*swish*	3000
Barley	42	*swish*	3200	40	*swish*	2600
Fresh maize (intact kernels)	37	*swish*	3400	16	*tanh*	3600
